# Multi-biomarker disease activity score as a predictor of disease relapse in patients with rheumatoid arthritis stopping TNF inhibitor treatment

**DOI:** 10.1371/journal.pone.0192425

**Published:** 2018-05-23

**Authors:** Marjan Ghiti Moghadam, Femke B. G. Lamers-Karnebeek, Harald E. Vonkeman, Peter M. ten Klooster, Janneke Tekstra, Annemarie M. Schilder, Henk Visser, Eric H. Sasso, David Chernoff, Willem F. Lems, Dirk-Jan van Schaardenburg, Robert Landewe, Hein J. Bernelot Moens, Timothy R. D. J. Radstake, Piet L. C. M. van Riel, Mart A. F. J. van de Laar, Tim L. Jansen, on behalf of the Dutch National POET Collaboration

**Affiliations:** 1 Department of Rheumatology, Medisch Spectrum Twente, Enschede, The Netherlands; 2 Department of Psychology, Health & Technology, University of Twente, Enschede, The Netherlands; 3 Department of Rheumatology, Radboud University Medical Center, Nijmegen, The Netherlands; 4 Department of Rheumatology, University Medical Center Utrecht, Utrecht, The Netherlands; 5 Department of Rheumatology, Medical Centre Leeuwarden, Leeuwarden, The Netherlands; 6 Department of Rheumatology, Rijnstate, Arnhem, The Netherlands; 7 Crescendo Bioscience, Inc., South San Francisco, CA, United States of America; 8 Department of Rheumatology, VU University Medical Center, Amsterdam, The Netherlands; 9 Department of Rheumatology, Jan van Breemen Research Institute/Reade, Amsterdam, the Netherlands; 10 Department of Rheumatology, AMC Amsterdam, Amsterdam, the Netherlands; 11 Department of Rheumatology, Ziekenhuis Groep Twente, Hengelo, The Netherlands; 12 Department of IQ Healthcare, Radboud University Medical Center, Nijmegen, The Netherlands; 13 Department of Rheumatology, VieCuri Medical Center, Venlo, The Netherlands; University of Nebraska Medical Center, UNITED STATES

## Abstract

**Objective:**

Successfully stopping or reducing treatment for patients with rheumatoid arthritis (RA) in low disease activity (LDA) may improve cost-effectiveness of care. We evaluated the multi-biomarker disease activity (MBDA) score as a predictor of disease relapse after discontinuation of TNF inhibitor (TNFi) treatment.

**Methods:**

439 RA patients who were randomized to stop TNFi treatment in the POET study were analyzed post-hoc. Three indicators of disease relapse were assessed over 12 months: 1) restarting TNFi treatment, 2) escalation of any DMARD therapy and 3) physician-reported flare. MBDA score was assessed at baseline. Associations between MBDA score and disease relapse were examined using univariate analysis and multivariate logistic regression.

**Results:**

At baseline, 50.1%, 35.3% and 14.6% of patients had low (<30), moderate (30−44) or high (>44) MBDA scores. Within 12 months, 49.9% of patients had restarted TNFi medication, 59.0% had escalation of any DMARD and 57.2% had ≥1 physician-reported flare. MBDA score was associated with each indicator of relapse. At least one indicator of relapse was observed in 59.5%, 68.4% and 81.3% of patients with low, moderate or high MBDA scores, respectively (*P* = 0.004). Adjusted for baseline DAS28-ESR, disease duration, BMI and erosions, high MBDA scores were associated with increased risk for restarting TNFi treatment (OR = 1.85, 95% CI 1.00–3.40), DMARD escalation (OR = 1.99, 95% CI 1.01–3.94) and physician-reported flare (OR = 2.00, 95% 1.06–3.77).

**Conclusion:**

For RA patients with stable LDA who stopped TNFi, a high baseline MBDA score was independently predictive of disease relapse within 12 months. The MBDA score may be useful for identifying patients at risk of relapse after TNFi discontinuation.

## Introduction

Rheumatoid arthritis (RA) is a chronic inflammatory disease that can cause joint damage and physical disability [1]. Early detection of RA and the availability of biologic agents have markedly improved outcomes in these patients [2]. Many studies have shown that the use of combinations of conventional synthetic DMARDs (csDMARDs) and biological DMARDs (bDMARDS) such as tumor necrosis factor inhibitors (TNFi) is effective for reaching and maintaining a state of low disease activity (LDA) or remission [3–5]. Once LDA or remission has been reached, patients often continue their combination therapy indefinitely. This practice may lead to overtreatment, as recent studies suggest that in some RA patients the more expensive TNFi can be tapered or stopped [6,7]. However, before implementing this therapeutic strategy in routine care, a validated predictor of disease relapse would be desirable [8].

Several studies have explored predictors of successful TNFi discontinuation. Results varied considerably, possibly due to differences in population, design and definitions of success, but most studies identified deep remission or lower disease activity at the time of discontinuation as a predictor [9–11]. Rheumatoid factor (RF) positivity, shorter disease duration, non-smoking and normal body mass index (BMI) may also be associated with better outcomes [10,12]. Although these studies all found that some patients could discontinue TNFi treatment without flaring, it remains a challenge to accurately predict which patients may successfully discontinue treatment and which are at higher risk of disease relapse [13].

Studies of strategies for reducing DMARD treatment have mainly evaluated the predictive value of conventional clinical measures of disease activity and traditional biomarkers such as rheumatoid factor (RF) and anti-cyclic citrullinated peptide antibodies (ACPA) [10,12,14]. However, new biomarkers with interesting potential have become available. The multi-biomarker disease activity (MBDA) blood test measures 12 serum proteins to produce a score that has been clinically validated as a measure of disease activity in patients with RA [15–17]. MBDA scores have been shown to reflect current clinical disease activity and changes in disease activity over time, including treatment responses in RA patients treated with TNFi [18]. The MBDA score assesses the activity of underlying biologic pathways rather than external signs and symptoms and may therefore provide information that is complementary to clinical assessment [16]. The MBDA score was a more accurate predictor of radiographic progression than the 28-joint Disease Activity Score with C-reactive protein (DAS28-CRP) or CRP, and it is often elevated in patients with low clinical disease activity or low CRP [18–22].

Recently, the MBDA score and ACPA were shown to be predictors of relapse for RA-patients in stable remission when treatments with conventional DMARDs and/or biological DMARDs were tapered, and for some patients, subsequently stopped. Prediction was strongest when MBDA score and ACPA were combined [23]. This finding, in a study of drug tapering, suggests that the MBDA score may be capable of predicting the outcome of complete TNFi discontinuation. The aim of the present study was to examine the prognostic value of the MBDA score for disease relapse after discontinuation of TNFi in RA patients with stable LDA.

## Methods

### Patient cohort

Data used for these analyses were from the Dutch POET trial (the Netherlands Trial Register, number NTR3112) [24]. The study was approved by the Ethical Review Boards of all participating hospitals. Ethical approval for the study was granted by the Committee on Research involving Human Subjects, region Arnhem-Nijmegen (Commissie Mensgebonden Onderzoek regio Arnhem-Nijmegen) and local feasibility by all regional Ethical Committees. Patients were included from March 2012 to March 2014 and written informed consent was obtained from all patients. In this pragmatic, multicenter, open-label, randomized clinical trial, RA patients with stable LDA were randomized 2:1 to either stop or continue TNFi treatment. Patients were included from March 2012 to March 2014 and written informed consent was obtained from all patients. All participating patients were ≥18 years old, had RA according to ACR 1987 criteria, and had received TNFi treatment for at least one year prior to inclusion. Patients had stable LDA for at least six months, defined as either two DAS28 with erythrocyte sedimentation rate (DAS28-ESR) measurements <3.2 or a rheumatologist clinical impression of remission or stable low disease activity and at least one CRP measurement <10 mg/L in the six months prior to inclusion. Nearly all patients were receiving concomitant csDMARDs. In the 6 months prior to inclusion, no dosage changes were allowed for csDMARDs or corticosteroids.

In total, 531 patients were randomized to stop TNFi treatment in POET and followed for 12 months [24]. Concomitant treatment with csDMARDs was continued. If RA flared (DAS28-ESR ≥3.2 with a change in DAS28-ESR >0.6) [25], TNFi could be restarted at the discretion of the treating rheumatologist. Because the current study focused on the value of the MBDA score as a predictor of disease relapse after discontinuation of TNFi treatment, only data from patients randomized to the stop group were used. For the current analyses, baseline serum samples were available to measure MBDA scores for 439 of the 531 patients in the group that stopped TNFi treatment.

### Measurements

Patients were evaluated by their treating rheumatologist and rheumatology nurse at baseline and at least once every 3 months thereafter, for a period of one year. Baseline measures included: age, sex, weight, height, disease duration, medication use, rheumatoid factor (RF) and ACPA status, concomitant use of csDMARDs and, for this post hoc analysis, the MBDA score. Clinical measurements were performed at every scheduled or unscheduled visit and included the ESR (mm/h), CRP (mg/l), 28-joint tender joint count (TJC28), 28-joint swollen joint count (SJC28), and a patient-reported assessment of general health on a 100 mm visual analog scale (VAS-GH). These component measures were combined to calculate DAS28-ESR [26]. Physician-reported flares and all changes in medication were recorded throughout the study.

### Serum biomarker measurement and MBDA score calculation

Serum samples were stored at −40°C from time of preparation until transfer to Crescendo Bioscience (South San Francisco, CA, USA), where they were stored at −70°C or lower until biomarker concentration testing was performed in the Crescendo clinical laboratory, which is certified under the CMS Clinical Laboratory Improvement Amendments and accredited by the College of American Pathologists for determination of Vectra® DA scores. Biomarker concentrations were measured by electrochemiluminescence-based multiplexed immunoassays (Meso Scale Discovery, Rockville, MD, USA). The MBDA algorithm combines the concentrations of 12 biomarkers (vascular cell adhesion molecule-1 [VCAM-1], epidermal growth factor [EGF], vascular endothelial growth factor-A [VEGF-A], interleukin [IL]-6, tumor necrosis factor-receptor type 1 [TNF-RI], matrix metalloproteinase [MMP]-1, MMP-3, cartilage glycoprotein 39 [YKL-40], leptin, resistin, serum amyloid A [SAA], and CRP)[15] to generate the MBDA score on a scale of 1 to 100, with previously validated categories for low (<30), moderate (30 to 44) and high (>44) disease activity [16].

### Statistical analysis

Baseline demographic and disease-related characteristics were compared between the 439 patients with a baseline MBDA assessment and the 92 patients without an MBDA assessment using independent samples t-tests and Mann-Whitney U tests for normally and non-normally distributed continuous variables, and Pearson χ^2^ tests for categorical variables. Disease relapse was defined three ways, using the criteria of: 1) restarting TNFi treatment, 2) any DMARD medication escalation and 3) physician-reported flare. DMARD medication escalation was defined as restarting TNFi treatment or starting or increasing the dosing of any bDMARD or csDMARD (including corticosteroids) [24]. Baseline characteristics of patients who did and did not meet the different criteria for relapse within the 12-month follow-up period were first compared using univariate logistic regression analyses. Patients who dropped out before 12 months of follow-up without meeting a criterion for relapse were counted in this analysis with those who continued to have a response. For each criterion for relapse, the proportions of patients in the low (<30), moderate (30–44) and high (>44) MBDA score groups who relapsed were compared by univariate Pearson χ^2^ tests with Bonferroni adjustment for the number of comparisons (P < (0.05 / 3 =) 0.017). Additional sensitivity analyses were performed limited to those patients that were included based on two available DAS28 scores <3.2 in the six months prior to inclusion, those who met the inclusion criteria for stable LDA but had moderate DAS28-ESR at baseline, and those who were in remission at baseline (DAS28 scores <2.6). One-year relapse-free survival was examined for the low, moderate, and high MBDA score groups using Kaplan-Meier survival curves; patients who dropped out early without disease relapse were censored at the time of withdrawal. Between-group differences in survival were tested by pairwise log-rank tests, again with Bonferroni adjustment for the number of comparisons (P < 0.017). Based on the results of the univariate χ^2^ tests and Kaplan-Meier survival analyses, baseline MBDA scores were dichotomized as high (>44) vs. moderate-to-low (≤44). Cox proportional hazard regressions were used to estimate the hazard ratio (HR), which may be interpreted as a relative risk, of high vs. moderate-to-low MBDA score for the time to relapse. Next, univariate and multivariable logistic regression analyses were performed to evaluate the association between disease relapse within 12 months and high baseline MBDA score in terms of unadjusted odds ratios (ORs), ORs adjusted for baseline DAS28-ESR score, and ORs further adjusted for all other variables that were significantly (P < 0.05) associated with a relapse criterion in the univariate logistic regression analyses. Final sensitivity analyses were performed in which all patients with a missing visit (missing DAS28 score at 3, 6, 9, or 12 months) were counted as a flare on all flare criteria. All analyses were performed using SPSS version 22.

## Results

### Demographic and clinical data at baseline

From the 531 patients who were randomized to stop TNFi treatment in POET, baseline serum samples were available for MBDA testing for 439 patients. Among these patients, 356 (81.1%) were included based on at least two available DAS28 scores <3.2, and 83 (18.9%) were included based on the rheumatologist clinical impression of remission or stable low disease activity in combination with at least one available CRP value <10 mg/L. Baseline demographic and clinical data were similar between patients with or without a baseline MBDA sample (Table 1). Patients were typically older Dutch Caucasian females, with longstanding RF-positive, erosive RA. Most patients were receiving their first TNFi, with 51.3% of 439 patients receiving adalimumab, 40.1% receiving etanercept and 8.6% receiving infliximab, certolizumab or golimumab. Clinical disease activity was generally low, in accordance with study inclusion criteria, and 349 (79.5%) patients were in remission (DAS28-ESR <2.6) at baseline. Seventeen (3.9%) patients dropped out during the first 12 months of follow-up because of their own decision to drop out (n = 13) or presence of a comorbidity (n = 4).

**Table 1:** Baseline characteristics of POET patients grouped according to sample availability for MBDA testing.

Characteristic (N = 531)	MBDA sample (n = 439)	No MBDA sample (n = 92)	P
Female, n (%)	296 (67.4%)	66 (71.7%)	0.419
Age (yrs.), mean (SD)	59.8 (10.8)	61.7 (10.6)	0.137
Disease duration (yrs.), median (IQR)	10 (6–17)	9 (6–16)	0.535
BMI, mean (SD)	25.9 (4.3)	25.9 (4.0)	0.854
RF positive, n (%)	270 (67.3%)	58 (68.2%)	0.872
ACPA positive, n (%)	277 (69.1%)	55 (64.7%)	0.431
Erosive disease, n (%)	252 (62.8%)	53 (62.4%)	0.932
ESR, median (IQR)	9.0 (5–17)	9.5 (5–18)	0.638
CRP, median (IQR)	2 (1–5)	3 (1–5.8)	0.388
TJC28, median (IQR)	0 (0–1)	0 (0–0)	0.043
SJC28, median (IQR)	0 (0–0)	0 (0–1)	0.328
PGA, median (IQR)	20.7 (9.0–28.1)	20.4 (5.0–23.4)	0.455
DAS28-ESR	2.0 (0.8)	1.9 (0.7)	0.549
MBDA score, mean (SD)	30.2 (12.6)	-	-
Number of TNFi, n (%)			0.819
1^st^	379 (86.5%)	80 (87.0%)	
2^nd^	50 (11.4%)	11 (12.0%)	
3^rd^	9 (2.1%)	1 (1.1%)	
csDMARD, n (%)			0.581
Methotrexate	382 (87.0%)	77 (83.7)	
Other csDMARD	35 (8.0%)	8 (8.7%)	
No DMARD	22 (5.0%)	7 (7.6%)	

TNFi = tumor necrosis factor-alpha inhibitors; DAS28 = disease activity score in 28 joints; BMI = body mass index; RF = rheumatoid factor; ACPA = anti-cyclic citrullinated peptide antibodies; ESR = erythrocyte sedimentation rate; CRP = C-reactive protein; TJC28 = 28-joint tender joint count; SJC28 = 28-joint swollen joint count; PGA = patient global assessment; MBDA = multi-biomarker disease activity; csDMARD = conventional synthetic disease modifying anti-rheumatic drug; SD (standard deviation); IQR (interquartile range).

### Association between baseline MBDA score and disease relapse

Baseline MBDA scores were low (<30) in 220 (50.1%) patients, moderate (30−44) in 155 (35.3%) patients, and high (>44) in 64 (14.6%). Clinical disease activity at baseline, as measured with the DAS28-ESR, was low in 413 (94.1%) patients and moderate in 26 (5.9%) patients. Within 12 months, 219 patients (49.9%) had restarted TNFi treatment, 259 (59.0%) patients had escalated any DMARD, and 251 (57.2%) had experienced at least one physician-reported flare. There was no significant difference in the proportion of patients experiencing a relapse between those who were included based on available DAS28 scores and those who were included based on the rheumatologist clinical impression and the CRP value (TNFi restart: 51.4% vs. 43.4%, P = 0.188; medication escalation: 60.1% vs. 54.2%, P = 0.325; physician-reported flare: 57.3% vs. 56.6%, P = 0.911).

**Fig 1:**
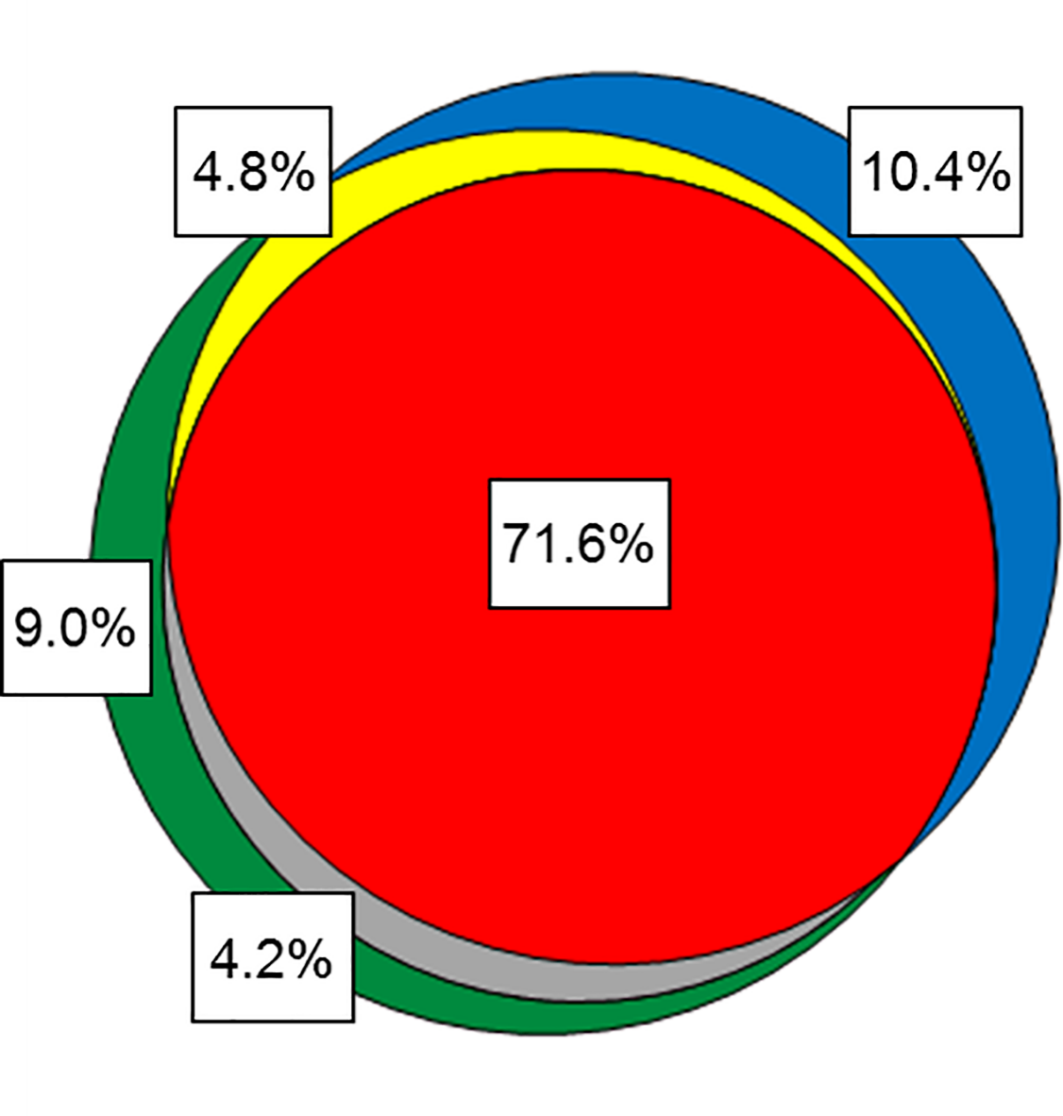
Venn diagram of patients meeting criteria for disease relapse. Red = TNFi restart; green = medication escalation; blue = physician-reported flare; yellow = overlap medication escalation / physician-reported flare; grey = overlap medication escalation / TNFi restart. Percentages are for the 289 patients who met at least one of the three criteria of disease relapse.

There was considerable overlap of the different relapse groups (Fig 1). For example, among the 289 patients who met at least one criterion for relapse, only 12 (4.2%) restarted TNFi treatment without also having a physician-reported flare, while 44 (15.2%) patients had a physician-reported flare but did not restart TNFi treatment; 207 out of 289 (71.6%) of the patients who relapsed met all three criteria. One hundred fifty (34.2%) patients in the overall cohort (N = 439) completed one year without meeting any of the three criteria for relapse.

High MBDA scores (>44) at baseline were univariately associated with significantly (Bonferroni adjusted P < 0.017) greater proportions of patients meeting the criteria for disease relapse (Table 2). At least one criterion of relapse was met within 12 months of TNFi discontinuation by 59.5%, 68.4% and 81.3% of patients with low, moderate, or high baseline MBDA scores, respectively (P = 0.004). Differences in the cumulative 12-month proportions of patients with relapse and the times to event were relatively small between patients with low or moderate MBDA scores, but patients with high MBDA scores were clearly at increased risk.

**Table 2:** Disease relapse by three criteria at 12 months for patients classified by baseline MBDA score.

Criterion for relapse	Total	Low (<30); n = 220	Moderate (30–44); n = 155	High (>44); n = 64	P
TNFi restart	219	102 (46.4%)	74 (47.7%)	43 (67.2%)	0.011
Medication escalation	259	117 (53.2%)	92 (59.4%)	50 (78.1%)	0.002
Physician-reported flare	251	116 (52.7%)	87 (56.1%)	48 (75.0%)	0.006
Any criterion	289	131 (59.5%)	106 (68.4%)	52 (81.3%)	0.004

Any criterion = TNFi re-initation, medication escalation, or physician-reported flare. P-value by Pearson χ^2^ test. Total N = 439.

Differences in disease relapse were very similar when limited to those patients that were included based on two available DAS28 scores <3.2 in the six months prior to inclusion (S1 Table), although no longer significant for TNFi restart after Bonferroni adjustment. Similar results were also obtained in a sensitivity analysis that excluded the 26 patients who met the inclusion criteria for stable LDA but had moderate DAS28-ESR at baseline (S2 Table). Among patients in remission at baseline (DAS28-ESR <2.6), differences between MBDA categories were slightly less pronounced and not significant after Bonferroni correction (S3 Table).

**Fig 2:**
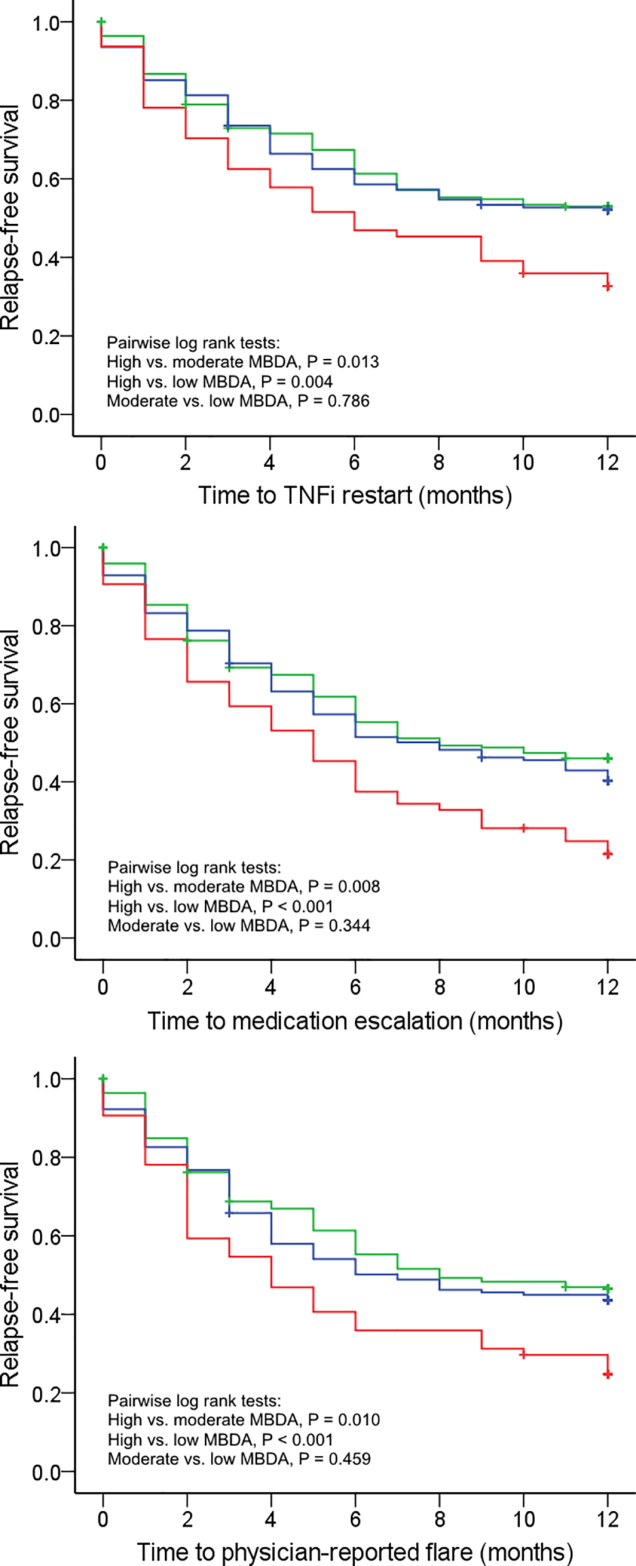
Kaplan-Meier survival curves. Low MBDA scores (<30, green; n = 220), moderate MBDA scores (30–44, blue; n = 155), high MBDA scores (>40, red; n = 64) for three definitions of disease relapse. TNFi restart (top), medication escalation (middle) and physician-reported flare (bottom).

Univariate Kaplan-Meier survival analyses confirmed the significantly decreased one-year relapse-free survival in patients with high baseline MBDA scores (Fig 2). Pairwise differences in one-year relapse-free survival were not significant between patients with low or moderate MBDA scores, but patients with high MBDA scores had significantly (Bonferroni adjusted P < 0.017) worse relapse-free survival than patients with either moderate or low MBDA scores for all three criteria of relapse. The HRs of high vs. moderate-to-low baseline MBDA scores were 1.61 (95% CI 1.15–2.25, P = 0.005) for TNFi restart, 1.66 (95% CI 1.22–2.26, P = 0.001) for medication escalation, and 1.64 (95% CI 1.20–2.25, P = 0.002) for physician-reported flare.

### Univariate and multivariate analysis of risk factors for disease relapse at 12 months

In univariate logistic regression analyses, greater disease duration was significantly associated with all three criteria for disease relapse at 12 months (ORs 1.04–1.05, 95% CI 1.01–1.08) (S4 Table). Higher BMI was associated with increased odds of restarting TNFi (OR 1.05, 95% CI 1.01–1.10), medication escalation (OR 1.07, 95% CI 1.02–1.13) and any criterion (OR 1.06, 95% CI 1.01–1.12). Erosive disease was significantly associated with restarting TNFi (OR 1.62, 95% CI 1.08–2.44), but not with the other two criteria. Baseline DAS28-ESR was significantly associated with medication escalation (OR 1.57, 95% CI 1.21–2.04), physician-reported flare (OR 1.36, 95% CI 1.06–1.75) and any criterion (OR 1.65, 95% CI 1.26–2.17), but not with restarting TNFi treatment (OR 1.25, 95% CI 0.98–1.60).

In multivariate analysis with adjustment for baseline DAS28-ESR, disease relapse after stopping TNFi treatment was more than twice as likely to occur in patients with a high baseline MBDA score, regardless of the relapse criterion used (Table 3). After adjusting for baseline DAS28-ESR, disease duration, BMI and erosions, high baseline MBDA score remained a significant independent predictor of disease relapse by all three criteria (Table 3). However, it did not remain significantly associated with the composite definition of relapse in the fully adjusted model. In a final sensitivity analysis in which all patients with a missing visit were counted as a flare, high MBDA score remained a significant predictor of clinical flare and a marginally significant predictor of medication escalation and TNFi-restart (S5 Table).

**Table 3:** Univariate and multivariate analyses of high (>44) versus moderate or low baseline MBDA score as a predictor of disease relapse at 12 months.

	Unadjusted		Adjusted		Fully adjusted	
Criterion for relapse	OR (95% CI)	P	OR (95% CI)	P	OR (95% CI)	P
**TNFi restart**						
MBDA >44	2.32 (1.32–4.05)	0.003	2.17 (1.23–3.83)	0.008	1.85 (1.00–3.40)	0.049
DAS28-ESR			1.17 (0.91–1.51)	0.219	1.10 (0.84–1.45)	0.474
Disease duration					1.05 (1.02–1.08)	<0.001
BMI					1.06 (1.01–1.11)	0.031
Erosive					1.30 (1.00–3.40)	0.248
**Medication escalation**						
MBDA >44	2.84 (1.52–5.31)	0.001	2.44 (1.29–4.62)	0.006	1.99 (1.01–3.94)	0.047
DAS28-ESR			1.47 (1.13–1.92)	0.004	1.48 (1.11–1.97)	0.008
Disease duration					1.04 (1.01–1.06)	0.011
BMI					1.07 (1.01–1.13)	0.014
Erosive					1.24 (0.79–1.97)	0.353
**Physician-reported flare**						
MBDA >44	2.54 (1.39–4.64)	0.002	2.31 (1.25–4.25)	0.007	2.00 (1.06–3.77)	0.033
DAS28-ESR			1.27 (0.98–1.65)	0.069	1.20 (0.92–1.58)	0.184
Disease duration					1.04 (1.01–1.06)	0.007
BMI					1.03 (0.98–1.08)	0.228
Erosive					1.08 (0.69–1.68)	0.784
**Any criterion**						
MBDA >44	2.52 (1.30–4.89)	0.006	2.12 (1.08–4.16)	0.029	1.68 (0.83–3.40)	0.147
DAS28-ESR			1.56 (1.18–2.07)	0.002	1.54 (1.14–2.07)	0.005
Disease duration					1.04 (1.01–1.07)	0.010
BMI					1.06 (1.00–1.12)	0.037
Erosive					1.25 (0.78–3.40)	0.347

DAS28-ESR, disease duration and BMI were analyzed as continuous variables; MBDA score (>44) and erosive (yes/no) were analyzed as categorical variables. Adjusted = adjusted for DAS28. Fully adjusted = adjusted for DAS28, disease duration, BMI and erosions. Any criterion includes patients with TNFi re-initation, medication escalation or physician-reported flare. Total N = 439. See Table 2 for n-values by relapse criterion.

## Discussion

Our analyses of the POET study show that, for RA patients in remission or stable LDA, a high MBDA score at the time of TNFi discontinuation was significantly associated with disease relapse during the next 12 months. Over 80% of patients with a high baseline MBDA score relapsed according to at least one of the three criteria we used. This rate of relapse was substantially higher for patients with low or moderate MBDA scores, suggesting that patients with low clinical disease activity and high MBDA scores may have inflammation that is partly or entirely subclinical. Several other studies have found the MBDA score to be frequently elevated when conventional clinical measures indicate remission or LDA [19,20,22,23]. Moreover, such patients were at increased risk for progressive joint damage [19,20,22]. Consequently, discontinuation of TNFi treatment in POET may have allowed a resurgence of residual inflammation and subsequent clinical relapse [23]. Our finding that the MBDA score was a predictor of relapse independently of DAS28-ESR suggests that it may be a clinically useful tool for identifying patients who are at increased risk of unsuccessful TNFi discontinuation.

Although high MBDA was an independent predictor of three predefined criteria for relapse (TNFi restart, medication escalation, and physician-reported flare), it should be noted that it did not remain significantly associated with meeting any criterion of flare when adjusting for all other potential predictors, including DAS28 score. Previous studies have explored predictors of disease relapse after TNFi discontinuation and, although results varied considerably, greater clinical disease activity at the time of discontinuation has been identified as a predictor [9–11]. Likewise, our study found that higher baseline DAS28-ESR scores were associated with two criteria for disease relapse, but not with the criterion of restarting TNFi treatment.

In other studies, RF positivity, shorter disease duration, non-smoking and normal body mass index (BMI) have been associated with successful TNFi discontinuation [10,12]. In the current study, higher BMI scores were univariately associated with increased odds of meeting two criteria of disease relapse but not physician-reported flare, and longer disease duration was a strong predictor for all three definitions of disease relapse. Erosive disease was univariately associated with TNFi restart. Neither positivity for RF nor ACPA was associated with disease relapse.

We previously demonstrated that RA patients in remission or stable low disease activity, as defined by the DAS28-ESR, had a relapse risk of approximately 50% within 12 months of discontinuing TNFi treatment in the POET study [24]. With such a high likelihood of relapsing, it may be helpful to have an effective tool to predict the outcome of treatment withdrawal. The MBDA score has been shown to correlate significantly with the DAS28-ESR, DAS28-CRP, simplified disease activity index (SDAI) and clinical disease activity index (CDAI), both overall and in seronegative and seropositive RA patients [16,27,28]. The MBDA score was an independent predictor of disease relapse in a study of RA patients in clinical remission who tapered, and in one arm of the study stopped, all RA therapy, including csDMARDs and bDMARDs [18]. The present study is the first to demonstrate the utility of the MBDA score as a predictor for risk of disease relapse in RA patients who discontinued TNFi treatment at baseline.

Patients with high MBDA had an odds ratio of approximately 2 for experiencing a relapse as defined by the three criteria. However, the proportion of patients with low or moderate MBDA scores who still relapsed within 12 months was also quite high, which may limit the utility of the MBDA to guide TNFi discontinuation in clinical practice. Additionally, since odds ratios tend to overestimate the probability of frequent events (the overall prevalence of relapse ranged from approximately 50%–60% on the different criteria), the hazard ratios or relative risks of around 1.6 found in the one-year survival analyses may be a more appropriate and precise estimate of risk for relapse associated with high MBDA.

Our study has several strengths. The data we analyzed were collected in POET, the largest randomized controlled trial on stopping TNFi in RA patients in remission or stable low disease activity to date. The MBDA score was measured in an unbiased selection of 439 patients in the stop group, comprising 83% of those who stopped TNFi treatment in POET. Most patients in our study had long disease duration (i.e., established RA) and an average age of 60 years, which is representative of the TNFi-using RA population in The Netherlands. Our finding that MBDA score was a predictor of relapse risk is strengthened by our having used 3 different definitions of disease relapse: 1) restarting TNFi treatment, 2) escalation of any DMARD therapy and 3) physician-reported flare, which identified more relapses than with any one criterion alone. MBDA scores at baseline were predictive of each definition of disease relapse.

A limitation of the study is that MBDA score was measured only at baseline. Longitudinal measurements may have provided insight into the effect of TNFi discontinuation on MBDA scores over time, and the potential for early change in MBDA score to predict relapse. Although only the MBDA score has been validated for measuring disease activity, it remains to be explored if any of the 12 biomarkers in the MBDA score might individually have ability to predict disease relapse.

In conclusion, for RA patients in remission or stable LDA, a high baseline MBDA score was frequently observed and was found to be an independent predictor of disease relapse within 12 months of TNFi discontinuation. These results suggest that the MBDA score may be a clinically useful tool for identifying subgroups of patients who have an increased risk of relapse when stopping TNFi treatment. These data should be confirmed in other studies.

## Supporting information

S1 Table Disease relapse by three criteria at 12 months for patients classified by baseline MBDA score in those patients included based on at least two available DAS28 scores <3.2 in the six months prior to inclusion. (DOC)

S2 Table Disease relapse by three criteria at 12 months for patients classified by baseline MBDA score excluding 26 patients with DAS28 ≥3.2 at baseline. (DOC)

S3 Table Disease relapse by three criteria at 12 months for patients classified by baseline MBDA score for patients in DAS28 remission at baseline. (DOC)

S4 Table Univariate associations between baseline variables and three definitions of disease relapse within 12 months in the stop group. (DOC)

S5 Table Univariate and multivariate analyses of high (>44) versus moderate or low baseline MBDA score as a predictor of disease relapse at 12 months. Sensitivity analysis with all patients with a missing visit (missing DAS28 score at 3, 6, 9 or 12 months) counted as a flare on all flare criteria.
(DOC)
