# Supplementary material for: Multi-biomarker disease activity score as a predictor of disease relapse in patients with rheumatoid arthritis stopping TNF inhibitor treatment
Source: PLoS One. 2018 May 23;13(5):e0192425. doi: 10.1371/journal.pone.0192425 (PMC5965880; doi:10.1371/journal.pone.0192425)
Supplement: S1 Table — (DOC) [file pone.0192425.s001.doc]

**Supplementary Table 1.** Disease relapse by three criteria at 12 months for patients classified by baseline MBDA score in those patients included based on at least two available DAS28 scores <3.2 in the six months prior to inclusion

| **Criterion for relapse** | **Total** | **Low (<30)**  **n=214** | **Moderate (30–44)**  **n=145** | **High (>44)**  **n=54** | **P** |
| --- | --- | --- | --- | --- | --- |
| TNFi restart | 183 | 88 (47.3%) | 62 (51.2%) | 33 (67.3%) | 0.044 |
| Medication escalation | 214 | 101 (54.3%) | 75 (62.0%) | 38 (77.6%) | 0.011 |
| Physician-reported flare | 204 | 98 (52.7%) | 69 (57.0%) | 37 (75.5%) | 0.016 |
| Any criterion | 234 | 111 (59.7%) | 84 (69.4%) | 39 (79.6%) | 0.019 |

Any criterion = TNFi re-initation, medication escalation, or physician-reported flare. P-value by Pearson χ^2^ test. Total N=356.
