# Supplementary material for: Multi-biomarker disease activity score as a predictor of disease relapse in patients with rheumatoid arthritis stopping TNF inhibitor treatment
Source: PLoS One. 2018 May 23;13(5):e0192425. doi: 10.1371/journal.pone.0192425 (PMC5965880; doi:10.1371/journal.pone.0192425)
Supplement: S2 Table — (DOC) [file pone.0192425.s002.doc]

**Supplementary Table 2.** Disease relapse by three criteria at 12 months for patients classified by baseline MBDA score excluding 26 patients with DAS28 ≥3.2 at baseline

| **Criterion for relapse** | **Total** | **Low (<30)**  **n=214** | **Moderate (30–44)**  **n=145** | **High (>44)**  **n=54** | **P** |
| --- | --- | --- | --- | --- | --- |
| TNFi restart | 205 | 99 (46.3%) | 69 (47.6%) | 37 (68.5%) | 0.012 |
| Medication escalation | 240 | 113 (52.8%) | 86 (59.3%) | 41 (75.9%) | 0.008 |
| Physician-reported flare | 236 | 113 (52.8%) | 82 (56.6%) | 41 (75.9%) | 0.009 |
| Any criterion | 269 | 127 (59.3%) | 99 (68.3%) | 43 (79.6%) | 0.012 |

Any criterion = TNFi re-initation, medication escalation, or physician-reported flare. P-value by Pearson χ^2^ test. Total N=413.
