# Supplementary material for: Multi-biomarker disease activity score as a predictor of disease relapse in patients with rheumatoid arthritis stopping TNF inhibitor treatment
Source: PLoS One. 2018 May 23;13(5):e0192425. doi: 10.1371/journal.pone.0192425 (PMC5965880; doi:10.1371/journal.pone.0192425)
Supplement: S3 Table — (DOC) [file pone.0192425.s003.doc]

**Supplementary Table 3.** **Disease relapse by three criteria at 12 months for patients classified by baseline MBDA score for patients in DAS28 remission at baseline**

| **Criterion for relapse** | **Total** | **Low (<30)**  **n=220** | **Moderate (30–44)**  **n=155** | **High (>44)**  **n=64** | **P** |
| --- | --- | --- | --- | --- | --- |
| TNFi restart | 170 | 88 (46.6%) | 55 (46.2%) | 27 (65.9%) | 0.065 |
| Medication escalation | 196 | 100 (52.9%) | 67 (56.3%) | 29 (70.7%) | 0.114 |
| Physician-reported flare | 191 | 97 (51.3%) | 64 (53.8%) | 30 (73.2%) | 0.038 |
| Any criterion | 217 | 109 (57.7%) | 77 (64.7%) | 31 (75.6%) | 0.078 |

Any criterion = TNFi re-initation, medication escalation, or physician-reported flare. P-value by Pearson χ^2^ test. Total N=349.
