# Supplementary material for: Multi-biomarker disease activity score as a predictor of disease relapse in patients with rheumatoid arthritis stopping TNF inhibitor treatment
Source: PLoS One. 2018 May 23;13(5):e0192425. doi: 10.1371/journal.pone.0192425 (PMC5965880; doi:10.1371/journal.pone.0192425)
Supplement: S4 Table — (DOC) [file pone.0192425.s004.doc]

**Supplementary Table 4.** Univariate associations between baseline variables and three definitions of disease relapse within 12 months in the stop group

| **Characteristic** | **TNFi restart** | **Medication**  **escalation** | **Physician-reported flare** | **Any flare** |
| --- | --- | --- | --- | --- |
| Female sex | 0.93 (0.63-1.39); 0.735 | 0.86 (0.57-1.29); 0.452 | 0.95 (0.63-1.42); 0.799 | 1.01 (0.66-1.53); 0.976 |
| Age | 1.00 (0.98-1.02); 0.990 | 1.01 (0.99-1.03); 0.242 | 1.01 (0.99-1.03); 0.170 | 1.01 (0.99-1.03); 0.300 |
| Disease duration | **1.05 (1.02-1.08); <0.001** | **1.04 (1.01-1.06); 0.003** | **1.04 (1.01-1.06); 0.003** | **1.04 (1.01-1.07); 0.003** |
| BMI | **1.05 (1.01-1.10); 0.030** | **1.07 (1.02-1.13); 0.005** | 1.04 (0.99-1.09); 0.137 | **1.06 (1.01-1.12); 0.018** |
| RF positive | 1.14 (0.75-1.74); 0.530 | 1.23 (0.80-1.88); 0.340 | 1.04 (0.68-1.58); 0.865 | 1.14 (0.73-1.77); 0.564 |
| Anti-CCP positive | 1.06 (0.70-1.62); 0.775 | 1.00 (0.65-1.55); 0.988 | 1.31 (0.86-1.01); 0.209 | 0.99 (0.63-1.56); 0.977 |
| Erosive disease | **1.62 (1.08-2.44); 0.020** | 1.46 (0.96-2.20); 0.075 | 1.31 (0.87-1.97); 0.195 | 1.50 (0.98-2.30); 0.060 |
| DAS28-ESR | 1.25 (0.98-1.60); 0.074 | **1.57 (1.21-2.04); 0.001** | **1.36 (1.06-1.75); 0.017** | **1.65 (1.26-2.17);<0.001** |
| MBDA score | **1.02 (1.01-1.04); 0.008** | **1.03 (1.02-1.05); <0.001** | **1.02 (1.01-1.04); 0.002** | **1.04 (1.02-1.05);<0.001** |
| Number of TNFi | 1.12 (0.71-1.76); 0.620 | 1.05 (0.66-1.67); 0.825 | 1.42 (0.88-2.29); 0.154 | 1.31 (0.79-2.16); 0.301 |
| Concomitant DMARD | 0.83 (0.35-1.96); 0.670 | 0.68 (0.29-1.61); 0.381 | 1.09 (0.45-2.60); 0.852 | 0.74 (0.31-1.77); 0.495 |

Values are for OR (95% CI) and P-value; boldfaced when P <0.05. Age, disease duration, BMI, DAS28-ESR and MBDA score were analyzed as continuous variables. All others were analyzed as categorical variables. N=439.
