# Supplementary material for: Multi-biomarker disease activity score as a predictor of disease relapse in patients with rheumatoid arthritis stopping TNF inhibitor treatment
Source: PLoS One. 2018 May 23;13(5):e0192425. doi: 10.1371/journal.pone.0192425 (PMC5965880; doi:10.1371/journal.pone.0192425)
Supplement: S5 Table — Sensitivity analysis with all patients with a missing visit (missing DAS28 score at 3, 6, 9 or 12 months) counted as a flare on all flare criteria. (DOC) [file pone.0192425.s005.doc]

**Supplementary Table 5.** **Univariate and multivariate analyses of high (>44) versus moderate or low baseline MBDA score as a predictor of disease relapse at 12 months. Sensitivity analysis with all patients with a missing visit (missing DAS28 score at 3, 6, 9 or 12 months) counted as a flare on all flare criteria.**

|  | **Unadjusted** | | **Adjusted** | | **Fully adjusted** | |
| --- | --- | --- | --- | --- | --- | --- |
| **Criterion for relapse** | **OR (95% CI)** | **P** | **OR (95% CI)** | **P** | **OR (95% CI)** | **P** |
| TNFi restart  MBDA >44  DAS28-ESR  Disease duration  BMI  Erosive | 2.13 (1.15–3.94) | 0.016 | 1.90 (1.02–3.55)  1.32 (1.01–1.72) | 0.044  0.040 | 1.82 (0.93–3.56)  1.32 (0.99–1.77)  1.07 (1.03–1.10)  1.02 (0.97–1.07)  1.26 (0.80–2.00) | 0.083  0.059  <0.001  0.443  0.318 |
| Medication escalation  MBDA >44  DAS28-ESR  Disease duration  BMI  Erosive | 2.64 (1.30–5.35) | 0.007 | 2.24 (1.09–4.60)  1.52 (1.14–4.60) | 0.029  0.004 | 2.04 (0.93–4.45)  1.58 (1.15–2.17)  1.06 (1.03–1.10)  1.04 (0.99–1.10)  1.33 (0.81–2.17) | 0.074  0.004  <0.001  0.129  0.256 |
| Physician-reported flare  MBDA >44  DAS28-ESR  Disease duration  BMI  Erosive | 2.50 (1.26–4.95) | 0.009 | 2.21 (1.10–4.42)  1.35 (1.03–1.79) | 0.025  0.033 | 2.07 (1.01–4.26)  1.32 (0.98–1.78)  1.05 (1.02–1.08)  1.00 (0.95–1.06)  1.00 (0.62–1.60) | 0.048  0.064  0.001  0.916  0.996 |
| Any criterion  MBDA >44  DAS28-ESR  Disease duration  BMI  Erosive | 2.62 (1.21–5.68) | 0.015 | 2.20 (1.00–4.83)  1.55 (1.14–2.10) | 0.050  0.005 | 1.88 (0.83–4.26)  1.54 (1.11–2.14)  1.06 (1.02–1.10)  1.04 (0.98–1.10)  1.34 (0.81–2.23) | 0.131  0.011  0.001  0.209  0.257 |

DAS28-ESR, disease duration and BMI were analyzed as continuous variables; MBDA score (>44) and erosive (yes/no) were analyzed as categorical variables. Adjusted = adjusted for DAS28. Fully adjusted = adjusted for DAS28, disease duration, BMI and erosions. Any criterion includes patients with TNFi re-initation, medication escalation or physician-reported flare. Total N=439.
